# Recycling Local Waste Glass Bottles into Cement Paste: Effect on Hydration, Microstructure, and CO_2_ Emission

**DOI:** 10.3390/ma16186195

**Published:** 2023-09-13

**Authors:** Fengming Ren, Xiwen Zhang, Mingxin Lin, Qing Wang, Jing Sun

**Affiliations:** 1 School of Civil Engineering, Guangzhou University, Guangzhou 510006, China; rfm@gzhu.edu.cn (F.R.); zxw@e.gzhu.edu.cn (X.Z.); 2 Panyu Dashi Construction Engineering Limited Company, Guangzhou 511430, China; djc1988@djc1988.com

**Keywords:** waste glass powder, cement paste, hydration, microstructure, CO_2_ emission

## Abstract

Large amounts of waste glass are generated along with the manufacturing of glass products, causing detrimental effects on the environment. Through crushing and ball-milling, waste glass powder (WGP) can be acquired from glass bottles and has been suggested in cementitious systems due to its potential pozzolanic activity. To better understand the impact of WGP on cementitious composites, experimental tests of rheology, heat of hydration, and strength development were conducted on cement pastes with and without WGP. Results show that the rheological performance of cement paste is improved when WGP with particles passing through 80 μm sieves is incorporated. The retarding effect and pozzolanic reaction were observed through X-ray diffraction patterns and thermo-gravimetric parameter analyses. A calcium hydroxide (CH) content calculation further confirms the secondary reactivity of WGP in cement pastes. Compared with the samples without WGP, the normalized CH content of binder per unit mass containing 35% WGP decreased by 21.01%, 24.94%, and 27.41% at the ages of 1, 28, and 90 days, respectively, which contributes to late-age strength development of pastes. At the same time, the hydration per unit of cement was increased by 21.53%, 15.48%, and 11.68%, which improved the cement efficiency. In addition, WGP particles provide nuclei for hydration products, facilitating the subsequent growth of C-S-H and strength development in late ages. Based on value engineering analysis, WGP was found to reduce the impact of Portland cement on the environment by 34.9% in terms of carbon dioxide emissions, indicating a bright prospect for WGP in the cement industry.

## 1. Introduction

Glass materials are widely used in daily life and industrial applications due to their stable chemical properties and aesthetic appearance. Large amounts of waste glass are generated along with the manufacturing and application of various glass products, such as waste glass bottles. The annual production of waste glass is about 21.23 million tons in China, whereas the recycling rate is merely 30.4%, causing occupation of land resources and pollution of adjacent environments due to its unbiodegradable nature [1]. Thus, rational utilization of waste glass has become an important issue for glass factories and waste glass collectors.

The main components of waste glass are generally acknowledged as amorphous SiO_2_, accounting for 70% or more [1,2]. This attracts researchers and engineers to take advantage of the pozzolanic activity in producing greener cementitious materials due to the fact that cement production is ecologically detrimental in terms of carbon dioxide (CO_2_) emissions. For instance, in 2020, cement clinker production reached 1.579 billion tons on the Chinese mainland alone, and the emissions of CO_2_ accompanying it reached over 1.23 billion tons, taking up 12.43% of the total CO_2_ emissions in all industries. Aiming at mitigating the increasingly tense greenhouse effect, specific schemes for the strategy of carbon neutrality and peak emissions have been deployed, advocating new patterns for high-quality/sustainable cement development. The most effective way is to reduce the amount of silicate cement clinker by incorporating a high volume of supplementary cementitious materials (SCMs) [3,4], such as fly ash [5], furnace slag [6], and high-reactivity metakaolin [7]. Herein, waste glass has also been proposed to partially substitute cement in concrete after mechanical grinding or chemical activation [8,9]. Utilizing waste glass in cementitious composites would definitely decrease the consumption of cement and lower the cost of relevant products, contributing to solid waste utilization and carbon emission mitigation [10,11,12,13].

A decrease in the particle size of WGP was reported to significantly increase the pozzolanic activity [14]; 75 μm was considered to be the largest diameter [15,16]. Before application, waste glass was mechanically ground into powder with different particle sizes according to specific requirements. WGP would participate in the secondary reaction of hydration thanks to its high content of amorphous silica. No particular difference was detected in the secondary calcium silicate hydrates (C-S-H) in the micromorphologies of concrete containing WGP [14,17] and concrete with fly ash [18] or silica fume [14] based on scanning electron microscope (SEM) observations. Shao et al. [2] reported that concrete containing WGP exhibited higher strength in both early and late ages than that with fly ash. Schwarz and Neithalath [19] compared the non-evaporated water content of cementitious composites with fine WGP and fly ash, indicating high pozzolanic activity of WGP in the system. Yusuf et al. [20] investigated the synergistic effect of WGP and silica fume and showed that the synergistic action of WGP and SF enhances the reorganization of silicates, making the binder more amorphous and reducing hydroxyl compounds, but morphologically causing heterogeneity in the microstructure. The authors also studied the residual strength and density of glass-adulterated concrete at elevated temperatures (550 °C). It was found that glass enhances the workability of concrete, shortens the setting time, reduces the density, and increases the residual strength and density [21]. In addition, feasibility studies have been conducted on the optimization of cementitious composites with various amounts of WGP to obtain high performance and high durability [10,22,23].

Thus, recycling waste glass bottles into WGP to produce a greener cementitious composite seems practicable. However, the effect of WGP from local waste glass bottles on the hydration and microstructure of cement paste has not been fully understood, especially for WGP with similar particle sizes to cement. Whether the rheological and mechanical properties of pastes containing different amounts of local WGP could meet relevant standards will be critically investigated for further application. Therefore, it is necessary and of great significance to study the effects of recycled WGP from waste glass bottles on the fresh and hardened properties of cementitious composites. Experimental tests of WGP content on the performance variation of cement pastes were carried out accordingly. Mineral compositions from an X-ray diffractometer (XRD) and microstructure morphologies under scanning electronic microscopy (SEM) were obtained to analyze the impact of WGP in substitution with cement. The ecological effect, focusing on CO_2_ emissions, was quantified correspondingly.

## 2. Experimental Program

### 2.1. Materials

**Figure 1:**
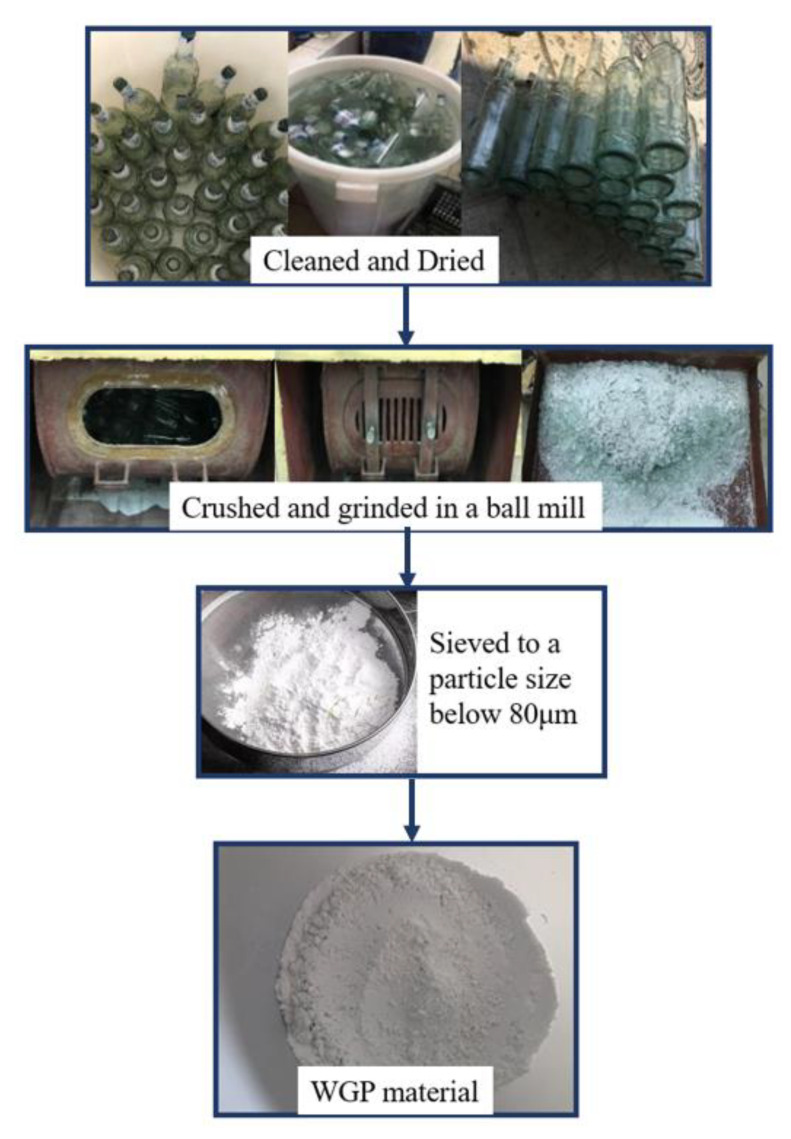
Preparation of WGP from local waste glass bottles.

**Figure 2:**
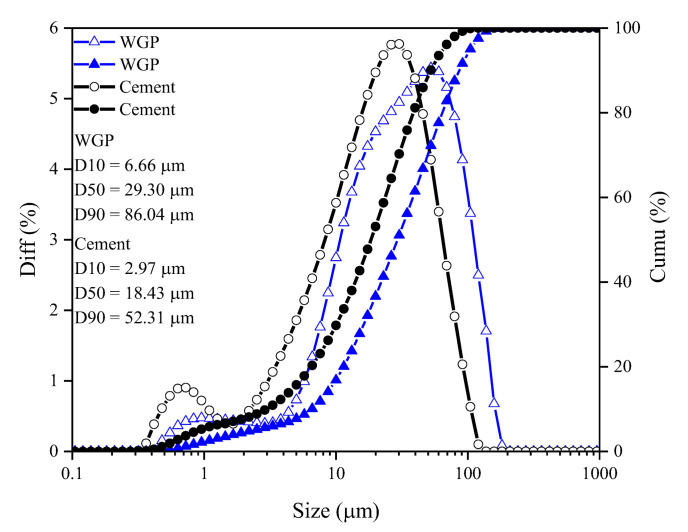
Particle size distribution of WGP and cement.

Common white-type waste glass bottles were provided by a local waste collection station in Guangzhou, China. Those bottles were first cleaned and dried, then crushed and ground in a ball mill to powder, as schematically shown in Figure 1. After sieving, WGP with a similar size to P.O. 42.5 Portland cement (as per Chinese Standard GB175 [24]) was prepared. The particle size distributions of cement and WGP are displayed in Figure 2, which were analyzed by a laser particle size analyzer MS2000 from Malvern Panalytical, UK. They share the same size range of 0.1 μm to 110 μm, facilitating the substitution of WGP for cement without drastically changing the grading of grains. The diameter of WGP (D10, D50, and D90 as 6.66 μm, 29.29 μm, and 96.04 μm) is slightly larger than that of cement (D10, D50, and D90 as 2.97 μm, 18.43 μm, and 52.31 μm). The apparent densities of cement and WGP are 3.10 g/cm^3^ and 2.51 g/cm^3^, respectively.

**Figure 3:**
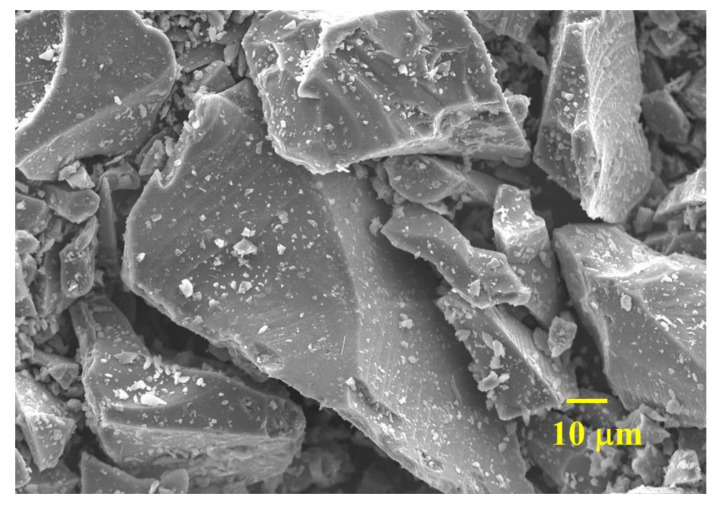
Micromorphology of WGP used (×1000).

The chemical compositions of both materials are listed in Table 1, tested by X-ray fluorescence (XRF) via PANalytical Axios XRF-1800. The main oxides of WGP are SiO_2_ (64.90 wt.%), CaO (19.62 wt.%), and Na_2_O (10.06 wt.%), implying a typical soda-lime silica glass and an alkaline glass material. Such a high content of SiO_2_ (mostly amorphous silica) would reveal pozzolanic activity based on previous research and related standards [25,26,27]. Figure 3 displays the micromorphology of WGP with its angular lumpy shape and irregular coarse surface, which might contribute to adequate contact with cement hydration products.

### 2.2. Mix Design and Specimen Preparation

Different substitution ratios of WGP for cement are proposed by British standard BS EN 196-1 [28], international standard ASTM C311 [29], and Chinese standard GB/T 12957 [30], 20%, 25%, and 30% in weight, respectively. In addition, BS EN 197-1 [4] also requires that the upper limit of pozzolanic material dosage be 35 wt.%. In terms of activity index, which is defined as the compressive strength ratio of samples with SCM to the control group, BS EN 450-1 [31] requires a value larger than 75% after curing for 28 days and 85% for 90 days. While in GB/T 2847 [32], the ratio becomes 65% at 28 days, and in ASTM C311 [29], it becomes 75% at 7 and 28 days.

To better illustrate the effect of local WGP on the properties of cement paste, the above weight percentages of 20%, 25%, and 30% were selected. In addition, 15 wt.% and 35 wt.% WGP replacements for cement were designed as contrasts. Detailed mix proportions with a constant water-to-cement ratio of 0.4 are listed in Table 2. The mixes in all dosages (even the 35% WGP content) showed good flow and no water secretion or segregation. The mixing procedure was consistent with Chinese standard GB/T 17671 [33]. Specimens with a size of 40 × 40 × 40 mm^3^ were prepared for compressive strength evaluation at the ages of 7, 28, and 90 days. All specimens were demolded after film covering for 24 h, then moved to a standard curing chamber at 20 ± 2 °C and ≥98% relative humidity until the test ages. After the compression tests were conducted at an MTS hydraulic universal testing machine, samples were carefully prepared from the core of the cubes for further microstructural analysis.

### 2.3. Test Methods

#### 2.3.1. Rheology

A rheometer with a co-axial cylindrical rheometer was applied to investigate the rheological parameters of all pastes. The shear speed was first increased from 5 to 100 revolutions per minute (RPM) in a step of 5 RPM, maintained for 15 s till the peak. Then, the shear speed was decreased at the same interval [34]. The Bingham model [35] was adopted to determine the rheological parameters.

#### 2.3.2. Heat of Hydration

The heat evolution of all mixtures was obtained via the TAM air isothermal calorimeter. According to the equilibrium principle of specific heat capacity, Equation (1) was proposed to calculate the amount of cement and free water at a constant quartz sand quantity ($m_{S}$) of 15 g.

(1) $$m_{S}c_{S}=m_{C}c_{C}+m_{W}c_{W}$$

where $m_{C}$ denotes the mass of cement and $m_{W}$ denotes the mass of water. Herein, the specific heat capacity of quartz sand $c_{S}$ is 0.76 J/gK, cement $c_{c}$ is 0.75 J/gK, and water $c_{W}$ is 4.18 J/gK. To analyze the impact of WGP, 15% and 30% WGP were applied to replace Portland cement by weight. Before testing, the calorimeter was first turned on and calibrated for three days. The samples were preweighed and mixed inside a standard ampoule for 2 min. Thereafter, the ampoules were placed inside the isothermal calorimeter and sealed with metal taps. The temperature of the instrument was set constantly to 25 °C, and real-time heat flows were detected and recorded for 7 whole days (168 h). 

#### 2.3.3. Microstructure Analysis

XRD analysis was performed to study the physical phase composition of cement pastes with and without WGP. Operating conditions were set as 40 kV, 100 mA, and Cu target in the diffractometer of the Japan Ultima type VI. The continuously scanning range was from 5° to 60°, with an average speed of 10°/min.

Thermo-gravimetric and differential thermal analysis (TG-DTA) experiments were carried out on the NETZSCH STA 2500. The temperature range was 30 °C to 1000 °C at a rate of 10 °C/min under a dynamic N_2_ atmosphere (20 mL/min).

The micromorphologies of hydration products were photographed by an SNE-4500 M Plus electron microscope. The instrument voltage was 15 kV, and thin layers of gold were sprayed on the surface of paste samples in advance to guarantee good conductivity for concrete specimens.

## 3. Results and Discussions

### 3.1. Rheological Properties

**Figure 4:**
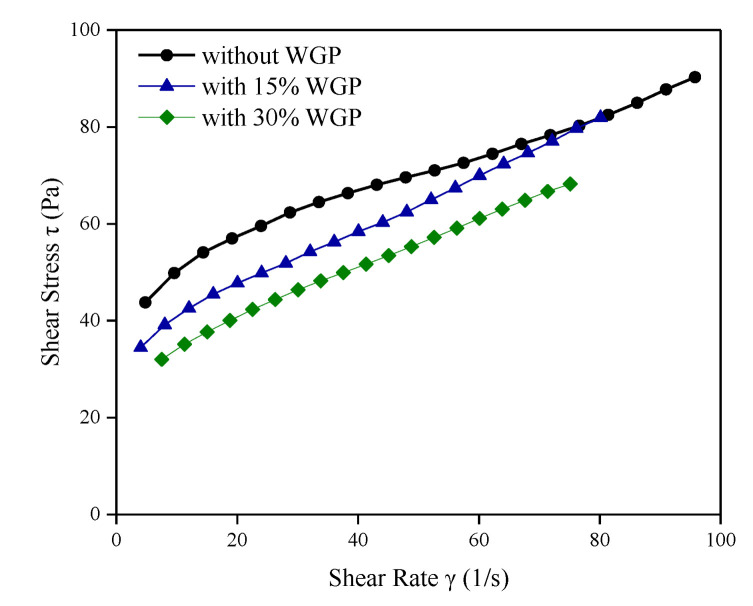
Shear stress-rate curves of fresh cement paste.

Shear stress-rate curves of fresh cement pastes with 15 wt.% WGP and 30 wt.% WGP are displayed in Figure 4 together with the control group. As expected, flowability varies in the presence of WGP, which is characterized by 90% grains smaller than 86μm and a relatively coarse surface. Shear stress of plain paste is higher than that with WGP addition, indicating a positive effect of WGP on workability, as also observed in [36]. When WGP content increases, shear stress decreases at the same shear rate, implying better flowability in paste with 30 wt.% WGP. It is attributed to the reduction in water demand because of the decrease in cement amounts when WGP is incorporated. Thus, more free water is released, facilitating the grain particles easy flow in the cementitious system. Another possible reason for the improvement in rheological performance lies in the appropriate grading of WGP particles. As reported by Lu et al. [37], large WGP particles (D50 ≥ 204 μm) show an adverse effect on fluidity due to the high friction of individual irregularly shaped particles, while small WGP particles (diameter less than 45 μm) also fail to improve flowability because of their high specific surface area and effective water demand [14,38]. Thus, a suitable WGP size range or proper ball-milling parameters for waste glass bottles should be duly considered in the design of cementitious composites with favorable flowability. WGP particles with sizes similar to cement grains were experimentally proven to be effective in improving the rheological performance in the present study.

### 3.2. Hydration

**Figure 5:**
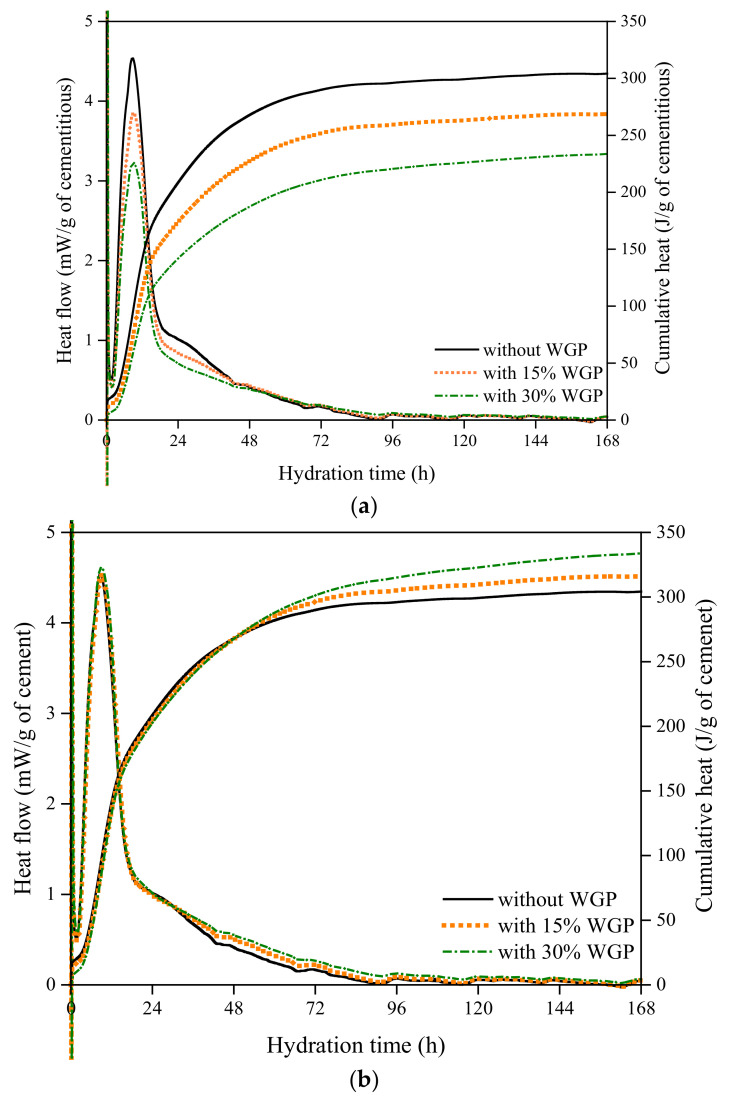
Heat evolution curves: (**a**) normalized heat evolution per unit binder in mass; (**b**) normalized heat evolution per unit cement mass.

Heat evolution in the first 168 h of plain paste, 15 wt.%, and 30 wt.% WGP-modified pastes is plotted in Figure 5. Two hydration parameters were analyzed: one is the heat flow per unit binder mass, and the other is the per unit cement mass (Figure 5b). Figure 5a reveals that the incorporation of WGP greatly decreases the heat flow rate and cumulative heat. Low activity of WGP at early ages is responsible for this diminishing trend, manifested by a lower heat evolution rate in pastes containing a higher amount of WGP. Such a retarding effect can also be reflected by the parameters of hydration peaks listed in Table 3. On the one hand, the hydration peaks appear later in WGP-modified pastes; 8.99 and 9.01 h in contrast with 8.69 h for the control. On the other hand, the intensity at the peaks decreases from 4.54 to 3.85 and 3.23 mW/g when 15% and 30% cement in weight are replaced by WGP. This retarding phenomenon in paste containing WGP from local waste glass bottles is similar to previous observations of other ground-glass powders [39,40]. The effective water-to-cement ratio increases when WGP is adopted, contributing to the delay of the hydration peak [41].

When heat evolution rate per unit cement is discussed (Figure 5b), higher cumulative heat is observed in the presence of WGP after 48 h, which is also confirmed by Mirzahosseini and Riding [41]. More cement grains are exposed to free water thanks to the low water demand of glass beads, which can be concluded to be the dilution effect of WGP [37,42]. The decrease in alkali content in WGP-modified paste with a lower cement amount is also responsible for the heat increment since a high alkali liquid solution helps to shorten the induction period and promote tricalcium silicate (C_3_S)/dicalcium silicate (C_2_S) dissolution and CH formation [43]. Along with the hydration process, WGP particles gradually dissolve and produce an alkaline composition, accelerating the hydration process after 48 h. Du et al. [44] suggested that the accelerating mechanism of WGP in hydration might be the adsorption of calcium ions and the provision of growth sites for C-S-H nucleation. Na_2_O in WGP serves as a catalyst for early C-S-H formation and development [45]. In addition, the filling effect of WGP with particle sizes less than 86μm would provide available space and surface for hydrate formation [46].

### 3.3. Strength Development

**Figure 6:**
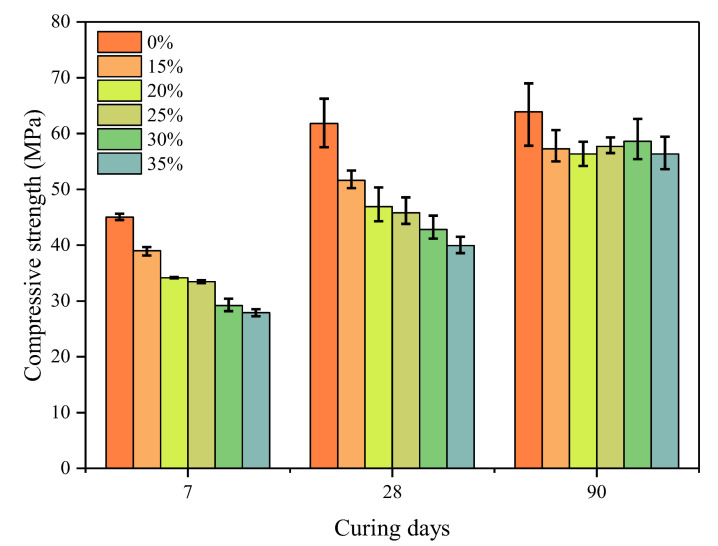
Compressive strength of pastes with and without WGP.

The cubic compressive strengths of all mixtures after standard curing for 7, 28, and 90 days are summarized in Figure 6. Strength decreases with an increment in WGP content, especially in the early ages. A reduction in cement amounts is responsible for this since hydration products are cut down. In the first seven days, the pozzolanic activity of WGP could not compensate for the decrease in strength due to the shortage of calcium hydroxide (CH). The average values of compressive strength after 90 days for WGP contents of 15–35 wt.% are close. Pastes containing 30 wt.% WGP exhibits the highest strength among all WGP-modified pastes, owing to its pozzolanic activity in the late hydration period [47]. This may be attributed to the pozzolanic reaction of WGP, which consumes CH in the cement paste, and the product generated fills the internal pores, thus reducing the strength loss rate.

**Figure 7:**
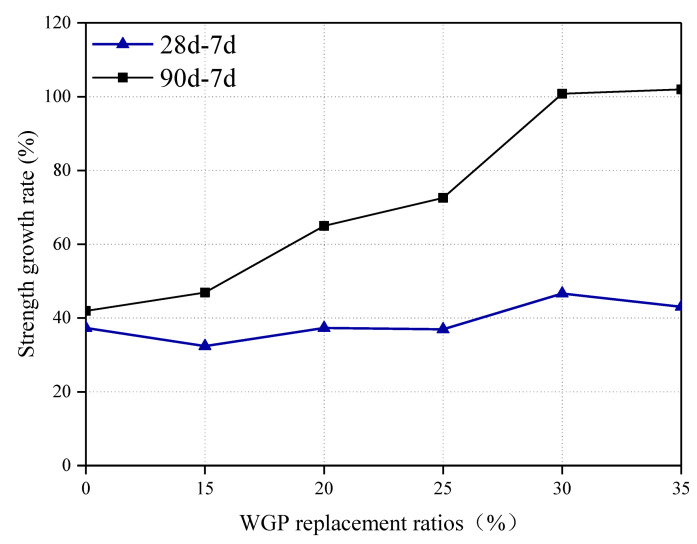
Strength growth rate of pastes with and without WGP.

Figure 7 further analyzes the strength growth rate of pastes with and without WGP. Herein, the strength growth rate is defined as the increase in the ratio of 28- or 90-day strength compared with 7-day strength. The growth rates in plain pastes at 28 and 90 days are close (37.3% and 41.9%), indicating a relatively weak development ability in strength. However, this situation is different for pastes containing WGP from waste glass bottles. Although the strength growth rate is lower at 28 days for paste with <25 wt.% WGP, larger values are observed for all WGP-modified pastes at 90 days. The ratios calculated even reach 100.80% and 101.96% for groups with 30 wt.% and 35 wt.% WGP, respectively. The secondary hydration reaction of WGP in late ages helps to improve mechanical properties, as also reported by other researchers [2,8].

As for the activity index of WGP, mainly three standards are quoted and compared. According to ASTM C311 [29], a 20% WGP replacement ratio is used, and its strength ratios at 7/28 days to plain pastes are calculated as 75.88% and 75.84%, slightly larger than the threshold of 75%. Based on BS EN 450-1 [31], the strength ratios of qualified pozzolanic WGP are 70% at 28 days and 80% at 90 days, with a WGP content of 25%. Herein, the average strengths of 25% WGP pastes are 45.79 and 57.70 MPa at 28 and 90 days, while plain cement pastes are 61.82 and 63.89 MPa. Ratios of 74.08% and 90.31% for 28 and 90 days meet the requirements of BS EN 450-1. When WGP increases to 30 wt.%, the activity index at the age of 28 days is evaluated as 69.23%, larger than the 65% required by GB 2847 [32]. Overall, WGP produced from waste glass bottles in the present study meets the basic requirement of pozzolanic materials for use in the cement industry in terms of activity index.

### 3.4. XRD Analysis

**Figure 8:**
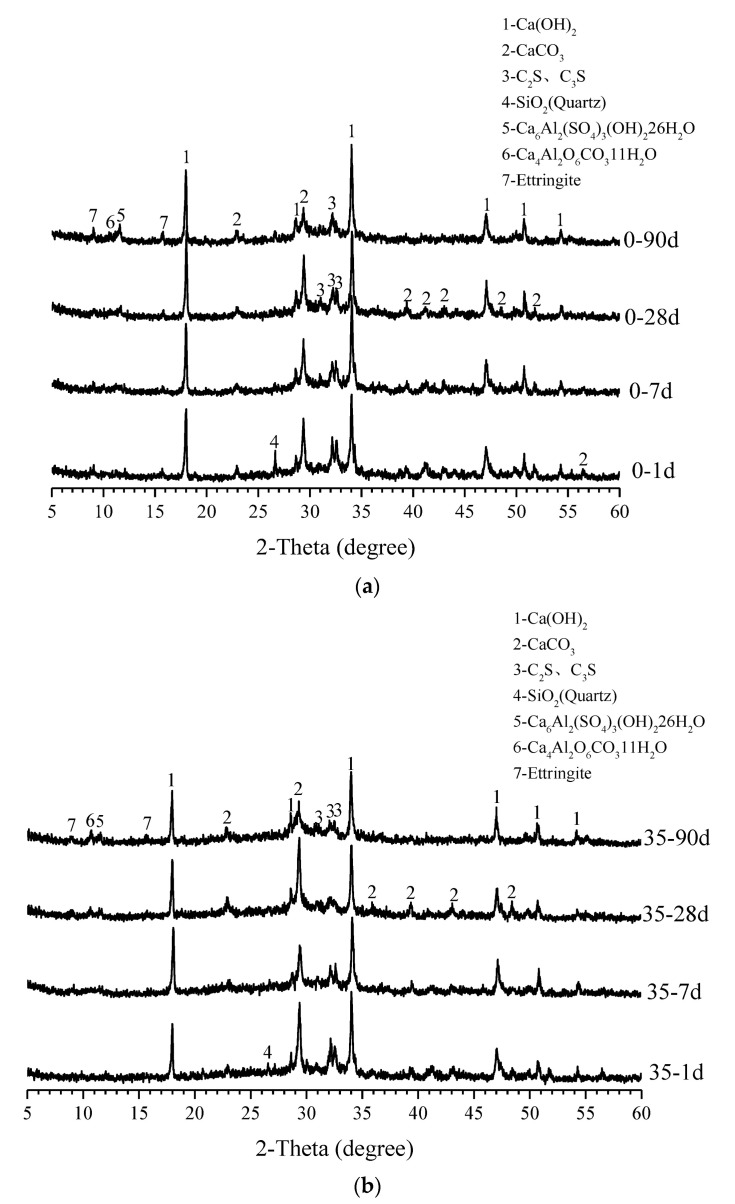
XRD patterns of (**a**) plain paste and (**b**) 35 wt.% WGP-modified paste.

XRD patterns of samples from plain pastes and 35 wt.% WGP-modified pastes at ages of 1, 7, 28, and 90 days are plotted in Figure 8. The main components in both pastes are CH, calcium carbonate (CaCO_3_), dicalcium silicate (C_2_S), tricalcium silicate (C_3_S), quartz (SiO_2_), ettringite (3CaO·Al_2_O_3_·3CaSO_4_·32H_2_O), and other minerals. The quartz SiO_2_ with diffraction peaks at an age of one day may be the impurity fraction contained in the cement itself. The hydration processes of C_3_S and C_2_S can be expressed in Equations (2) and (3), in which the hydration of C_2_S is a bit slower and gives off less heat under the same conditions [48].

(2) $$2C_{3}S+6H\to C_{3}S_{2}H_{3}\left(C-S-Hgel\right)+3CH+120cal/g$$

(3) $$2C_{2}S+4H\to C_{3}S_{2}H_{3}\left(C-S-Hgel\right)+CH+62cal/g$$

Herein, hyphens in C-S-H are used to represent the category of calcium silicate hydrated since the chemical composition of C-S-H is not constant and changes with hydration degree and original minerals. When hydration is completely accomplished, C-S-H can then be described as C_3_S_2_H_3_, suitable for stoichiometric calculations [49]. Activated silica could react with the above hydration product CH, called secondary hydration or pozzolanic reaction, as shown in Equation (4):

(4) $$CH+S\overset{}{\to }C-S-Hgel$$

Figure 8a displays the hydration products at various curing ages for plain paste. The peaks of C_2_S and C_3_S gradually decrease as the hydration process proceeds, while the intensity of the hydration product CH slightly increases. When recycled WGP is adopted, the hydration degree of cement becomes higher, as depicted in Figure 8b, because the differences in endmembers of C_2_S and C_3_S are even greater in comparison with the control group, indicating an accelerating effect of WGP. Meanwhile, CH diffraction peaks exhibit a weakening trend in pastes with WGP. Herein, at one day, the difference between CH peaks of paste with and without WGP is not obvious. However, after 90 days of curing, CH peaks in WGP-modified paste are much smaller than those in the control group, implying consumption of CH in the presence of WGP. As cement hydration proceeds, CH content increases and the concentration of hydroxyl ions increases, prompting the pozzolanic reaction of WGP with amorphous silica [47]. Once consumption of CH gets larger than its generation from cement hydration, the total amount decreases, resulting in a weak intensity of CH endmembers. Such a chemical reaction would produce more hydration products and enhance the microstructure of pastes, which well explains the strength improvement of WGP-modified pastes at a later age.

### 3.5. CH Content

**Figure 9:**
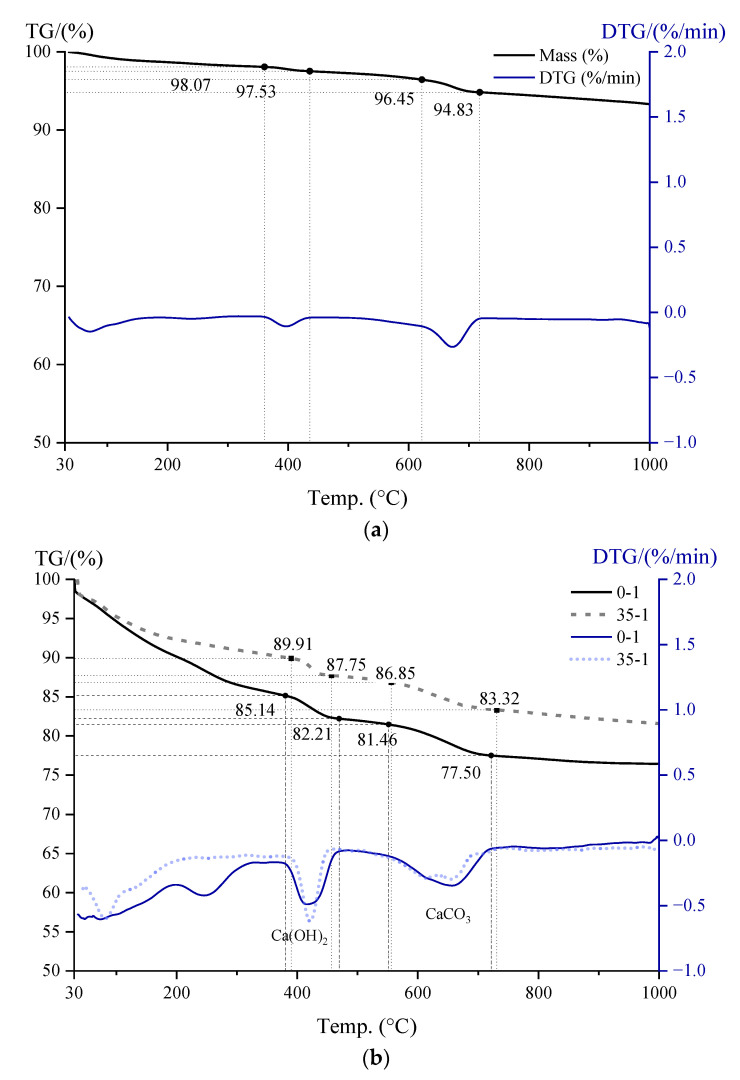

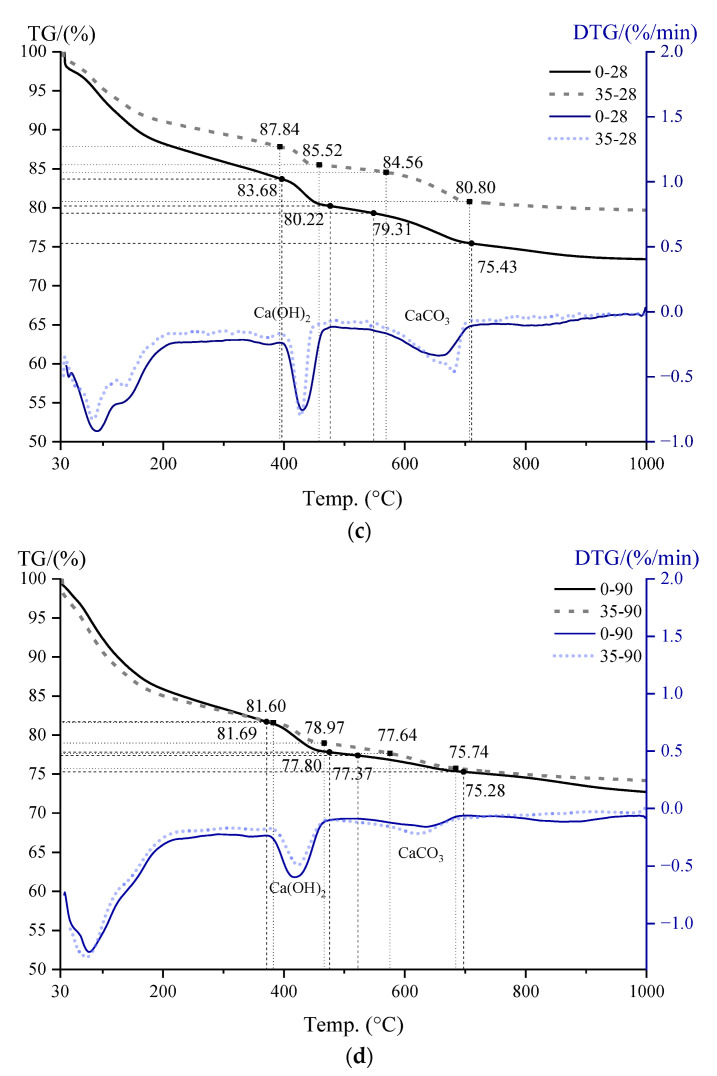
TG−DTG curves of the samples: (**a**) cement; (**b**) pastes at 1 day; (**c**) pastes at 28 days; (**d**) pastes at 90 days.

The pozzolanic activity of WGP could be reflected more specifically by the CH content change in pastes. Thermo-gravimetric parameters enable identification of physical phases and determination of CH content in hydrated pastes [48,50]. Based on the results of thermo-gravimetric tests (Figure 9a), cement itself contains CH and CaCO_3_. Therefore, in order to minimize the effect of CaCO_3,_ a modified calculation method is proposed. The measured CaCO_3_ content usually includes both raw material and CH carbonation, so it is essential to determine the CH and CaCO_3_ content of the raw cement. CaCO_3_ content measured typically includes both raw material and CH carbonation, which makes the determination of CH and CaCO_3_ in the original cement critically necessary. The TG and DTG curves of cement, plain paste, and 35 wt.% WGP-modified paste are displayed in Figure 9. Generally, the heat absorption peak around 400 is caused by the dehydration decomposition of CH and 650 °C by CaCO_3_. Based on the data from TG-DTG tests, the amount of CH can be quantitatively calculated as follows: The results are summarized in Table 4.

Firstly, for cement, the weight loss around 400 °C caused by CH dehydration is 98.07%−97.53%=0.54%. According to the chemical equation shown in Equation (5), the amount of CH is 0.54%(18/74)=2.22%, indicating a CH content of 2.22% in raw cement.

(5) $${Ca(OH)}_{2}\to CaO+H_{2}O(↑)\;\mathrm{Molecular weight (g/mol)}:\;\;74\;\;18$$

The weight loss around 650 °C caused by CaCO_3_ decomposition is 96.45%−94.83%=1.62%, based on Equation (6), 1.62%(44/100)=3.68% CaCO_3_ exists in raw cement.

(6) $${CaCO}_{3}\to CaO+{CO}_{2}(↑)\;\mathrm{Molecular weight (g/mol)}:\;\;100\;\;44$$

**Figure 10:**
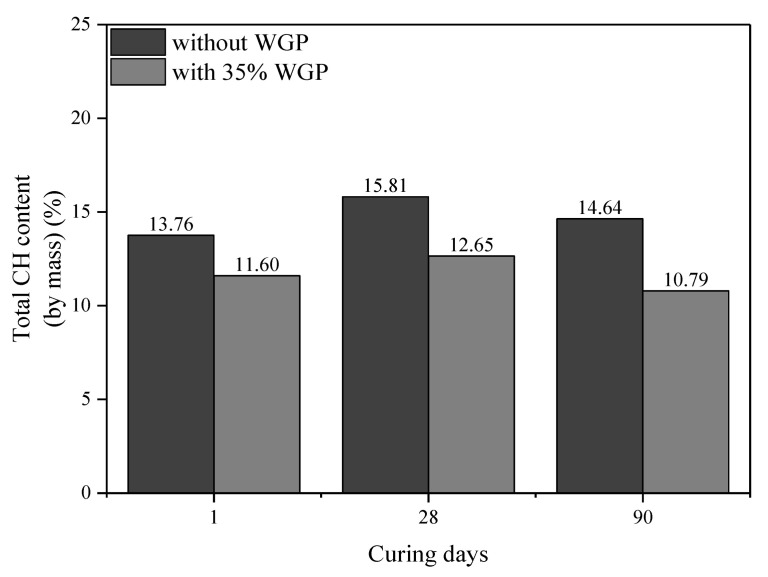
Total CH content of plain cement paste and 35% WGP-modified paste at different ages.

Secondly, Figure 9b shows the TG-DTG curves of plain paste and 35 wt.% WGP-modified paste after curing for 24 h. Similarly, the CH content of paste with WGP can be calculated as (89.91%−87.75%)(18/74)=8.88%, while the amount of CaCO_3_ is (86.85%−83.32%)(44/100)=8.02%. Since raw cement contains 3.68% CaCO_3_, the original amount of CaCO_3_ is 3.68%×(100%−35%)=2.39%, and the amount generated by CH carbonation is 8.02%−2.39%=5.63%. The CH content in this section is then 5.63%(100/74)=4.16%. On the basis of the 2.22% CH amount in the original cement, the total CH amount can be computed as 8.88%+4.16%−2.22%×(100%−35%)=11.60%. Figure 9c,d gives the total CH content of pastes with and without WGP at 28 and 90 days, summarized in Figure 10. CH content increases from 1 day to 28 days but decreases at 90 days for both series. Differences between pastes with and without WGP are 3.10%, 3.13%, and 3.87% for 1 day, 28 days, and 90 days, respectively, indicating evident pozzolanic activity of WGP in consuming CH in the samples, especially at later ages.

**Figure 11:**
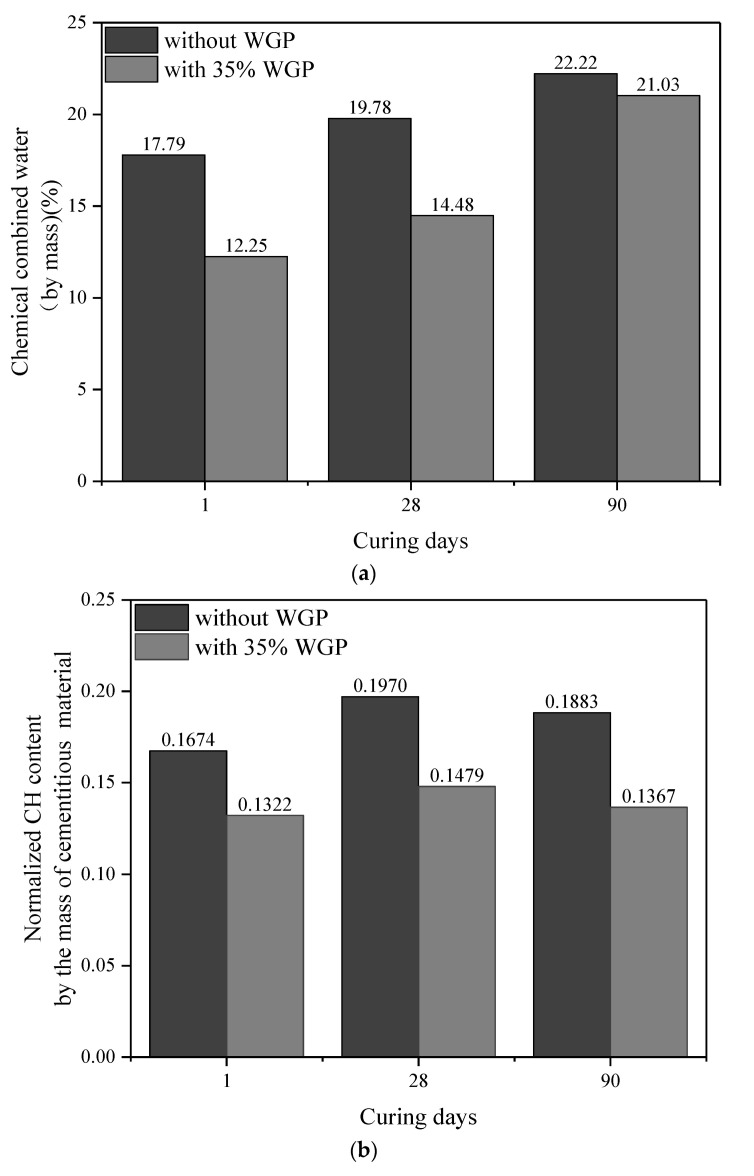

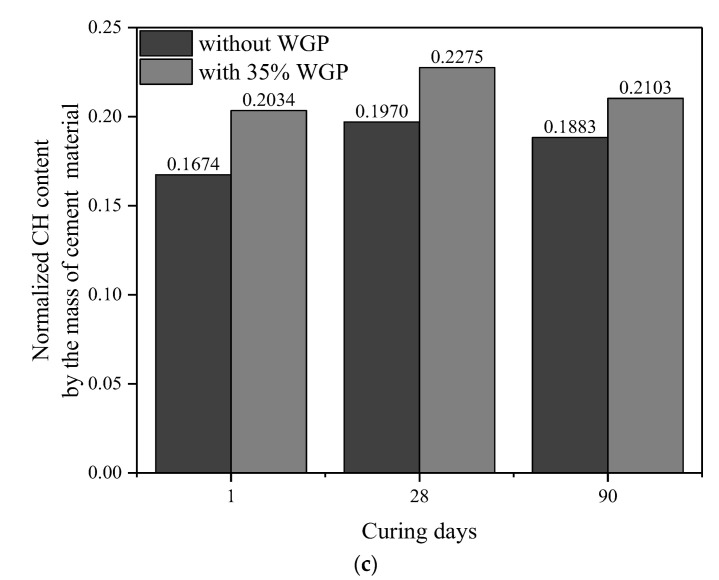
Amounts of chemical-bonded water and normalized CH content: (**a**) chemical-bonded water; (**b**) normalized CH content per unit binder mass; (**c**) normalized CH content per unit cement mass.

Thirdly, chemical-bonded water can be calculated right after decomposition of CH [51] as 100%−87.75%=12.25% for 35 wt.% WGP-modified paste curing for one day, which can reflect the hydration degree of cement indirectly. Thus, normalized CH content per unit binder mass is calculated as 11.60%(100−12.25)%=0.13, which means 1 g of binder produces 0.13 g of CH after one day of hydration. Furthermore, normalized CH content per unit cement mass can be worked out as 0.13(100%−35%)=0.20. Figure 11 displays the amounts of chemical-bonded water and normalized CH content. The amounts of chemical-bonded water in WGP-modified paste are lower than plain paste for all ages, whereas the growth rate is distinctly higher at 90 days. Compared with the samples without WGP, the CH content in the cementitious material containing 35% WGP decreased by 21.01%, 24.94%, and 27.41% at the curing ages of 1, 28, and 90 days, respectively. In addition, the normalized CH content per unit cement mass in Figure 11c confirms that WGP used can improve the hydration degree by 21.53%, 15.48%, and 11.68% at ages 1, 28, and 90 days, respectively, contributing to the late-age strength development of pastes.

### 3.6. Microstructure

**Figure 12:**
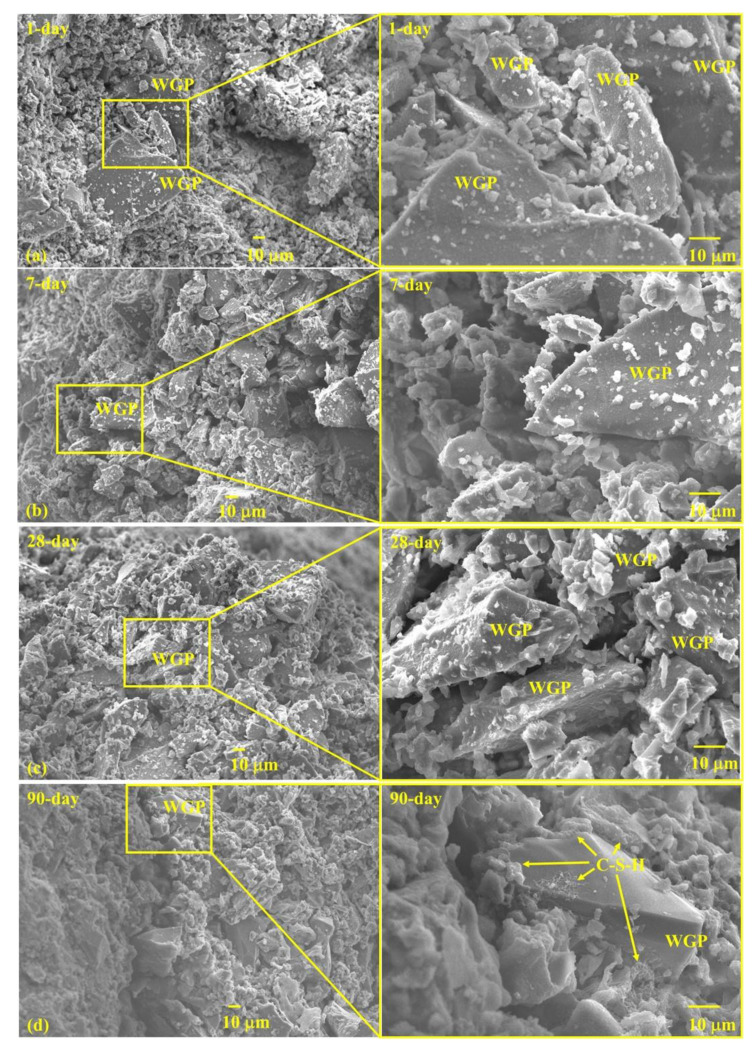
Micromorphologies of WGP-modified cement pastes: (**a**) 1 day; (**b**) 7 days; (**c**) 28 days; (**d**) 90 days.

Micromorphologies of pastes containing WGP from waste glass bottles are shown in Figure 12 with various curing ages. WGP particles with diameters ranging from 10 to 80 μm are clearly photographed after mixing for 24 h (Figure 12a). Several hydrates appear on the surface of WGP particles, which are probably the nuclei of C-S-H, as suggested by Ouyang et al. [52]. Figure 12b shows the paste morphology after curing for seven days. Herein, the amount of total hydration products increases and the microstructure gets denser, with WGP particles surrounded by discontinuous hydration products. After curing for 28 days, glass powder grains are almost wrapped by granular nuclei growing in hydrates. The structure of the paste becomes more compact, as reflected by an increase in the size and quantity of hydrate sites. Interval pores between WGP and cement particles get smaller compared to those in the earlier ages, implying higher resistance to external forces and liquid transportation. At 90 days, WGP particles are closely covered by C-S-H gels, resulting in less surface area in the same field of view. Active silica is responsible for the microstructure change since it dissolves in pore solution and reacts with CH accompanied by cement hydration [1]. WGP particles provide nucleation sites for C-S-H gel and other hydration products. Based on the analysis through the entire hydration process, such a pozzolanic reaction is weak at early ages but becomes stronger after curing for 28 days and more, resulting in dense microstructure and further enhances the long-term properties of pastes.

## 4. CO_2_ Emissions

The cement manufacturing process contributes greatly to CO_2_ emissions, accounting for nearly a quarter of total CO_2_ emissions [53]. WGP to replace Portland cement would lower the negative influence of cement-based materials on the environment. Herein, value engineering analysis (*V*) from the perspective of economics is adopted to analyze WGP impact by comparing the performance function (*F*) and carbon cost (*C*) of cement paste, expressed in Equation (7) [54].

(7) $$V=\frac{F}{C}$$

This study focuses on the function of 90-day compressive strength and the carbon cost during the whole process of pastes with and without WGP. Generally, CO_2_ emissions in the production of 1 ton of cement include those from raw material extraction, manufacturing, and transport (35.15 kg), decomposition (462.87 kg), calcination (505.92 kg), and product transportation (58.56 kg) [55]. To sum up, 1 ton of cement generates up to 1062.5 kg of CO_2_. For WGP recycled from local waste glass bottles by crushing, grinding, and sieving, the main equipment used for the recycling process is ball mills and sieving machines. The consumption of electricity is recorded as 3 kW·h per ton of WGP. When coal-fired power is assumed, CO_2_ production is 0.93 kg/(kW·h), which gives a CO_2_ emission of 2.79 kg, according to Cabrera and Wolley [56].

The cost and function of plain paste and pastes with 15%, 20%, 25%, 30%, and 35% WGP in weight are listed in Table 5. For the control group, 1367 kg of cement is required for 1 m^3^ of paste, and the emission of CO_2_ reaches 1452.44 kg. When the cement is partially substituted by WGP from glass bottles, the cost greatly decreases based on the above analysis of CO_2_ emissions. CO_2_ emissions are taken as the C, i.e., the cost of carbon. In this paper, the compressive strength function is taken as F. For instance, 35 wt.% WGP-modified paste costs 945.42 kg, merely 65.1% of the plain paste, reducing it by 34.9%. And the value (FC) increased from 0.044 to 0.060, increasing it by 26.7%. Although the incorporation of WGP may show a slight adverse effect on the strength function, a clear increasing trend in engineering value is detected, demonstrating the ecological effect of WGP in mitigating the emission impact of cement pastes. It shall be noted that the function here considers only the 90-day compressive strength of pastes; other parameters such as flexural strength, permeability, and carbonation resistance could be adopted for further analysis with higher accuracy.

## 5. Conclusions

In the present study, WGP recycled from local waste glass bottles is introduced in cement paste production. Its impact on the performance and carbon dioxide emissions of cement pastes were investigated. Based on experimental tests of rheology, heat of hydration, strength, and discussions of XRD, TG-DTG, and microstructure results, the following conclusions can be drawn:

- Recycling waste glass bottles into cement paste is technically feasible in terms of the mechanical properties required by relevant standards.
- WGP reduces the ecological impact of Portland cement by 34.9%, according to CO_2_ emission analysis via value engineering analysis.
- The addition of 35% WGP increased cement hydration by 21.53%, 15.48%, and 11.68% at curing ages of 1, 28, and 90 days, respectively, compared to without WGP.
- The pozzolanic reaction of WGP consumes CH in cementitious systems, as confirmed by XRD patterns and CH content analysis from TG-DTG curves. Compared with the samples without WGP, the normalized CH content per unit binder mass containing 35% WGP decreased by 21.01%, 24.94%, and 27.41% at the ages of 1, 28, and 90 days, respectively.
- WGP particles provide nucleation places for hydration products, facilitating subsequent growth of C-S-H and strength growth in late ages.

## Figures and Tables

**Table 1:** Chemical compositions and physical properties of WGP and cement used (wt.%).

Types	CaO	SiO_2_	Al_2_O_3_	Fe_2_O_3_	SO_3_	MgO	K_2_O	TiO_2_	SrO	MnO	Na_2_O	P_2_O_5_	Cl	ZnO	Rb_2_O	ZrO_2_	Density (g/cm^3^)	D50 (μm)
Cement	68.52	14.03	5.98	3.80	3.08	2.55	0.91	0.58	0.16	0.14	0.13	0.06	0.02	0.02	0.01	-	3.10	18.43
WGP	18.62	64.90	3.17	0.26	0.16	1.54	0.88	0.12	0.10	-	10.06	0.04	0.05	-	0.04	0.08	2.51	29.29

**Table 2:** Mix proportions of cement pastes with and without WGP (kg/m^3^).

Groups	Content		
	Cement	WGP	Water
Plain paste	1367.00	0.00	546.80
15% WGP	1161.95	205.05	546.80
20% WGP	1093.60	273.40	546.80
25% WGP	1025.25	341.75	546.80
30% WGP	956.90	410.10	546.80
35% WGP	888.55	478.45	546.80

**Table 3:** Mix proportions of cement pastes for heat hydration and parameters of hydration peaks.

Mixture	w/b	Water(g)	Cement (g)	WGP (g)	Hydration Peaks		Intensity of Hydration Peaks	
					Age (h)	Relative Value (%)	Intensity (mW/g)	Relative Value (%)
Plain paste	0.4	1.8827	4.7069	0.0000	8.69	1.000	4.54	1.000
15% GP	0.4	1.8827	4.0009	0.7060	8.99	1.035	3.85	0.848
30% GP	0.4	1.8827	3.2948	1.4121	9.01	1.037	3.23	0.711

**Table 4:** Normalized CH content summarized.

Type	Age (Days)	Original Content		Carbonation Reaction Content		Total CH (%)	Normalized CH	
		CH (%)	CaCO_3_ (%)	CaCO_3_ (%)	CH (%)		Per 1 g Binder	Per 1 g Cement
Cement	-	2.22	3.68	-	-	-	-	-
Plain paste	1	12.05	9.00	5.32	3.94	13.76	0.1674	0.1674
35% WGP	1	8.88	8.02	5.63	4.17	11.60	0.1322	0.2034
Plain paste	28	14.22	8.82	5.14	3.80	15.81	0.1970	0.1970
35% WGP	28	9.54	8.55	6.15	4.55	12.65	0.1479	0.2275
Plain paste	90	16.07	4.75	1.07	0.79	14.64	0.1883	0.1883
35% WGP	90	10.81	4.32	1.93	1.42	10.79	0.1367	0.2103

**Table 5:** Estimation of environmental CO_2_ emissions of pastes (1 m^3^).

Groups	Content			Cost	Function	Value
	Cement (kg)	WGP (kg)	Water (kg)	CO_2_ Emissions (kg)	Compressive Strength (MPa)	F/C
Plain paste	1367.00	0.00	546.80	1452.44	63.89	0.044
15% WGP	1161.95	205.05	546.80	1235.14	57.26	0.046
20% WGP	1093.60	273.40	546.80	1162.71	56.35	0.048
25% WGP	1025.25	341.75	546.80	1090.28	57.70	0.053
30% WGP	956.90	410.10	546.80	1017.85	58.61	0.058
35% WGP	888.55	478.45	546.80	945.42	56.36	0.060

## Data Availability

All data, models, and code generated or used during the study appear in the published article.
